# SSTAR, a Stand-Alone Easy-To-Use Antimicrobial Resistance Gene Predictor

**DOI:** 10.1128/mSphere.00050-15

**Published:** 2016-01-13

**Authors:** Tom J. B. de Man, Brandi M. Limbago

**Affiliations:** Centers for Disease Control and Prevention, Division of Healthcare Quality Promotion, Atlanta, Georgia, USA; University of Rochester

**Keywords:** antimicrobial resistance genes, porins, BLAST, SSTAR

## Abstract

We present the easy-to-use Sequence Search Tool for Antimicrobial Resistance, SSTAR. It combines a locally executed BLASTN search against a customizable database with an intuitive graphical user interface for identifying antimicrobial resistance (AR) genes from genomic data. Although the database is initially populated from a public repository of acquired resistance determinants (i.e., ARG-ANNOT), it can be customized for particular pathogen groups and resistance mechanisms. For instance, outer membrane porin sequences associated with carbapenem resistance phenotypes can be added, and known intrinsic mechanisms can be included. Unique about this tool is the ability to easily detect putative new alleles and truncated versions of existing AR genes. Variants and potential new alleles are brought to the attention of the user for further investigation. For instance, SSTAR is able to identify modified or truncated versions of porins, which may be of great importance in carbapenemase-negative carbapenem-resistant *Enterobacteriaceae*. SSTAR is written in Java and is therefore platform independent and compatible with both Windows and Unix operating systems. SSTAR and its manual, which includes a simple installation guide, are freely available from https://github.com/tomdeman-bio/Sequence-Search-Tool-for-Antimicrobial-Resistance-SSTAR-.

**IMPORTANCE** Whole-genome sequencing (WGS) is quickly becoming a routine method for identifying genes associated with antimicrobial resistance (AR). However, for many microbiologists, the use and analysis of WGS data present a substantial challenge. We developed SSTAR, software with a graphical user interface that enables the identification of known AR genes from WGS and has the unique capacity to easily detect new variants of known AR genes, including truncated protein variants. Current software solutions do not notify the user when genes are truncated and, therefore, likely nonfunctional, which makes phenotype predictions less accurate. SSTAR users can apply any AR database of interest as a reference comparator and can manually add genes that impact resistance, even if such genes are not resistance determinants *per se* (e.g., porins and efflux pumps).

## Abstract (precis)

Whole-genome sequencing (WGS) is quickly becoming a routine method for identifying genes associated with antimicrobial resistance (AR). However, for many microbiologists, the use and analysis of WGS data present a substantial challenge. We developed SSTAR, software with a graphical user interface that enables the identification of known AR genes from WGS and has the unique capacity to easily detect new variants of known AR genes, including truncated protein variants. Current software solutions do not notify the user when genes are truncated and, therefore, likely nonfunctional, which makes phenotype predictions less accurate. SSTAR users can apply any AR database of interest as a reference comparator and can manually add genes that impact resistance, even if such genes are not resistance determinants *per se* (e.g., porins and efflux pumps).

## INTRODUCTION

Antimicrobial resistance (AR) is an ancient phenomenon with a rapid increase in recent years and is a serious worldwide public health threat (1, 2). The emerging carbapenem-resistant microorganisms, like *Enterobacteriaceae* and *Pseudomonas*, are especially a concern since they are rendering many treatment options ineffective. The emergence of carbapenem resistance is mediated mainly by acquired carbapenemase genes that encode proteins which inactivate carbapenems and other β-lactam antibiotics (3). Other mechanisms, including modification or loss of outer membrane porin and efflux mechanisms, both of which can be combined with the production of extended-spectrum beta-lactamases (ESBLs) or AmpC-type β-lactamases, have also been described (4). AR genes can be acquired through horizontal gene transfer or can arise due to spontaneous mutations in chromosomal loci. Rapid identification of AR determinants, including prediction of novel alleles, is important for understanding the molecular epidemiology of AR pathogens and informing infection control interventions and may be useful for guiding patient therapy.

In recent years, the cost of DNA sequencing has been decreasing rapidly and the technique has become available for a large community worldwide (5). Whole-genome sequencing (WGS) data are used for strain characterization in the context of outbreak investigation, epidemiological surveillance, and the detection of genes that are associated with antimicrobial resistance (6 – 8). Many online databases containing AR genes are currently available to the public. The two most complete and up-to-date repositories are ResFinder (9) and the Comprehensive Antibiotic Resistance Database (CARD) (10). For these, users upload bacterial genome sequences to a Web service and subsequently retrieve a list of acquired antimicrobial resistance genes. These Web services are relatively slow, which can be a hurdle for users wanting to query multiple genome files.

Offline solutions, including SRST2 (11), ARG-ANNOT (12), and ABRicate (https://github.com/tseemann/abricate), exist as well. In addition to containing acquired resistance genes, the ARG-ANNOT database includes point mutations in chromosomal target genes known to be associated with AR. SRST2, ARG-ANNOT, and ABRicate run locally and give the user more control than Web-based services. SRST2 and ABRicate offer only a command-line interface, while ARG-ANNOT runs on an outdated software package (13). Currently, there is no freely available standalone AR gene detection tool with a “mouse click user interface.” While ARG-ANNOT and ABRicate offer a way to detect putative new AR genes, none of the above-mentioned tools offer an easy way to detect new or truncated alleles or variants of existing AR genes or other genes associated with resistance.

Here, we describe our tool SSTAR (Sequence Search Tool for Antimicrobial Resistance), which combines a modified ARG-ANNOT database, standalone BLAST (14), and an easy-to-use graphical user interface that enables the detection of known AR genes and predicts putative new variants as well as truncated genes in a fast, local, and easily updatable tool.

## RESULTS

### SSTAR algorithm.

**FIG 1:**
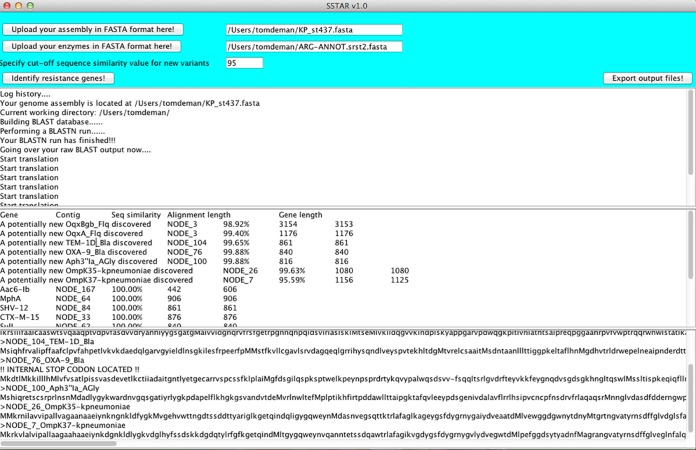
Graphical user interface of SSTAR. The top two buttons are used for uploading the genome assembly and AR gene collection. The user also fills out a sequence similarity cutoff value for detecting potential new variants.

SSTAR accepts two sequence files in FASTA format, one containing the bacterial genome assembly and the other the AR gene collection (Fig. 1). First, the genome assembly is turned into a local nucleotide BLAST database using the BLAST “makeblastdb” utility. Next, AR genes are queried against this reference using BLASTN. AR genes with 100% sequence similarity against 40% or more of a potential gene on the assembly are reported by SSTAR. Reports of more than one partial variant of the same gene located on the same contig suggest an assembly error or artifact, whereas multiple intact variants represent the presence of multiple alleles of the same gene.

If all BLASTN hits for a particular clustered AR gene group (e.g., NDM or OXA-48-like) have 95% to 99.99% sequence similarity, SSTAR reports a potentially new variant (PNV) of that gene. The complete region of alignment for each PNV is automatically selected and translated by SSTAR into protein sequence for all three reading frames. PNVs that span less than 80% of an AR gene will not be translated. Resistance determinants with <95% similarity to potential genes on the assembly are not reported.

Although we used a 95% threshold, this value can be set by the user to be more or less stringent for sequence similarity between genes in the AR gene database and those on the genome assembly. Here, we chose 95% since many AR genes within a gene group, like NDM and KPC, differ by only a few nucleotides from each other. When selecting lower similarity values (e.g., 30%), one is more likely to observe false-positive PNVs.

The translated sequence with the fewest internal stop codons is considered the output sequence. If the output protein sequence contains an internal stop codon, it will be marked as truncated. The user should then compare each translated PNV to sequences in the NCBI nonredundant (NR) database using BLASTP. Any potential novel beta-lactamase variant should be sent for confirmation and nomenclature to the NCBI at http://www.ncbi.nlm.nih.gov/projects/pathogens/submit_beta_lactamase.

### Resistance genes detected with SSTAR and ARG-ANNOT.

**FIG 2:**
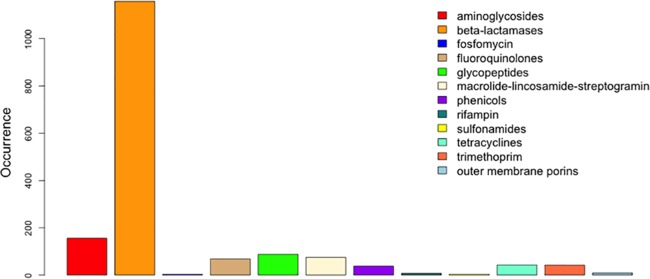
Composition of the modified SSTAR SRST2 ARG-ANNOT antimicrobial resistance database. We manually included wild-type outer membrane porin sequences for *Escherichia coli*, *Klebsiella pneumoniae*, *Enterobacter cloacae*, and *Enterobacter aerogenes* and excluded *tetR* and *bla*_OXA-30_.

We downloaded the SRST2 version of the ARG-ANNOT database from http://katholt.github.io/srst2/ and manually added several wild-type outer membrane porin sequences (see Materials and Methods). The compositions and distribution of antimicrobial resistance mechanisms in our modified database are illustrated in Fig. 2.

Genomic data generated from a *Klebsiella pneumoniae* ST437 isolate compared to our customized SRST2 ARG-ANNOT database using SSTAR identified numerous resistance mechanisms, including *bla*_NDM-5_, *bla*_SHV-12_, *bla*_OXA-232_, and *bla*_TEM-1_, as well as an intact *bla_ampH_* penicillin binding protein (15, 16) and a truncated *bla*_OXA-9_ gene (Table 1). SSTAR recognized an internal stop codon within *bla*_OXA-9_, caused by a single nucleotide polymorphism (SNP) and flagged the resultant output with a FASTA-ineligible message. Genes conferring resistance to other classes of antimicrobials, including aminoglycosides, fluoroquinolones, macrolides, sulfonamides, fosfomycin, and trimethoprim, were also identified in *K. pneumoniae* ST437 (Table 1).

**TABLE 1:** Resistance genes identified in *K. pneumoniae* ST437 and *E. coli* ST44 using the ResFinder 2.1 Web service or SSTAR software combined with customized versions of the SRST2 ARG-ANNOT and ResFinder databases

AR gene detected	Associated resistance	Presence or absence in *K. pneumoniae* ST437			Presence or absence in *E. coli* ST44		
		ResFinder 2.1	SSTAR/A^d^	SSTAR/R^e^	ResFinder 2.1	SSTAR/A	SSTAR/R
*aac(6′)-lb-cr*	Aminoglycosides^a^	–	–	–	+	+	+
*aac(6′)-lb*	Aminoglycosides	+	+	+	–	–	–
*aph(3′) la*	Aminoglycosides	+	+	+	–	–	–
*aadA1*	Aminoglycosides	+	–	+	–	–	–
*aadA2*	Aminoglycosides	–	–	–	+	+	+
*aadA5*	Aminoglycosides	–	–	–	+	+	+
*strA*	Aminoglycosides	–	–	–	+	+	+
*strB*	Aminoglycosides	–	–	–	+	+	+
*rmtF*	Aminoglycosides	+	+	–	–	–	–
*bla*_NDM-5_	Beta-lactams	+	+	+	–	–	–
*bla*_SHV-12_	Beta-lactams	+	+	+	–	–	–
*bla*_CTX-M-15_	Beta-lactams	+	+	+	+	+	+
*bla*_TEM-1_	Beta-lactams	+	+	+	–	–	–
*bla*_OXA-1_	Beta-lactams	–	–	–	+	+	+
*bla_OXA-9_*	Beta-lactams	+	+^b^	+^b^	–	–	–
*bla*_OXA-232_	Beta-lactams	+	+	+	–	–	–
*bla_ampH_*	Beta-lactams	–	+	–	–	+	–
*bla_ampC1_*	Beta-lactams	–	–	–	–	+	–
*bla_ampC2_*	Beta-lactams	–	–	–	–	+	–
*mph(A)*	Macrolides	+	+	+	+	+	+
*catB4*	Chloramphenicol	–	–	–	+	+	+
*sul1*	Sulfonamides	+	+	+	+	+	+
*sul2*	Sulfonamides	–	–	–	+	+	+
*fosA*	Fosfomycin	+	–	+	–	–	–
*tet(A)*	Tetracyclines	–	–	–	+	+	+
*tet(B)*	Tetracyclines	–	–	–	+	+	+
*dfrA12*	Trimethoprim	–	–	–	+	+	+
*dfrA14*	Trimethoprim	+	+	+	–	–	–
*dfrA17*	Trimethoprim	–	–	–	+	+	+
*oqxA*	Fluoroquinolones	+	+	+	–	–	–
*oqxB*	Fluoroquinolones	+	+	+	–	–	–
*qnrS1*	Fluoroquinolones	+	+	+	–	–	–
*E. coli* ompC (porin)^c^	Beta-lactams	–	–	–	–	+^b^	+^b^
*E. coli* ompF (porin)^c^	Beta-lactams	–	–	–	–	+^b^	+^b^
*K. pneumoniae ompK35* (porin)^c^	Beta-lactams	–	+	+	–	–	–
*K. pneumoniae ompK36* (porin)^c^	Beta-lactams	–	+	+	–	–	–
*K. pneumoniae ompK37* (porin)^c^	Beta-lactams	–	+	+	–	–	–

^a^ And fluoroquinolones.

^b^ A truncated variant was identified.

^c^ This sequence was manually added to the database.

^d^ SSTAR/A, SSTAR with SRST2 ARG-ANNOT.

^e^ SSTAR/R, SSTAR with SRST2 ResFinder.

A second analysis was performed on a carbapenem-resistant *Escherichia coli* ST44 isolate. The SSTAR ARG-ANNOT customized database identified resistance genes, including the beta-lactamase genes *bla*_OXA-1_ and *bla*_CTX-M-15_, as well as two additional putative cephalosporinase *bla_ampC_* genes that were present in the ARG-ANNOT database (GenBank accession numbers FN649414 and CP002970) and a *bla_ampH_* gene (Table 1). As with the *K. pneumoniae* isolate discussed above, genes conferring resistance to numerous classes of antimicrobials were identified in the genome of *E. coli* ST44, including aminoglycosides, fluoroquinolones, macrolides, chloramphenicol, sulfonamides, tetracyclines, and trimethoprim (Table 1).

### Comparison of resistance genes resulting in the observed resistance phenotype.

MIC values of all carbapenems and cephalosporins tested were elevated for both isolates (Table 2). The NDM carbapenemase identified in *K. pneumoniae* ST437 explains all beta-lactam resistance observed, with the exception of that to aztreonam, which is not hydrolyzed by metallo-beta-lactamases (17). However, this strain also carries genes that encode two ESBLs, *bla*_SHV-12_ and *bla*_CTX-M-15_, as well as another class D carbapenemase, *bla*_OXA-232_, all of which may contribute to aztreonam resistance. With *K. pneumoniae* ST437, elevated MICs of tobramycin, amikacin, and gentamicin were also observed, most likely due to the presence of *rmtF* (18) (Table 1). Resistance to the fluoroquinolones in *K. pneumoniae* ST437 is likely impacted by the presence of *qnrS1* and the *oqxA* and *oqxB* efflux pump genes (19, 20). No acquired resistance genes were identified to explain the elevated MICs of chloramphenicol or tetracyclines.

*E. coli* ST44 was not susceptible to any of the carbapenems tested and was resistant to all of the extended-spectrum beta-lactams, despite the lack of any known carbapenemase genes; *bla*_CTX-M-15_ was the only ESBL identified. *E. coli* ST44 was resistant to tobramycin but susceptible to amikacin and gentamicin, despite it carrying the aac*(6′)-lb-cr* aminoglycoside resistance element. This isolate also carried *aadA2*, *aadA5*, *strA*, and *strB*, which are expected to confer resistance to streptomycin and spectinomycin (21, 22). *Escherichia coli* ST44 was resistant to both ciprofloxacin and levofloxacin but had no acquired fluoroquinolone resistance determinants except *aac(6′)-lb-cr*. Genes encoding resistance to chloramphenicol (*catB4*) and tetracycline (*tetA* and *tetB*) were also observed in *E. coli* ST44, which likely explains its resistance to these two agents.

### Porin analysis.

Many phenotypically carbapenem-resistant *Enterobacteriaceae* do not produce a carbapenemase enzyme but instead harbor a combination of other resistance mechanisms to confer a resistance phenotype. Therefore, we examined the outer membrane porin genes in this organism, which have been described as contributing to carbapenem resistance in organisms that do not produce a carbapenemase (4, 23).

In order to interrogate the chromosomal porin genes in *E. coli* and *K. pneumoniae*, wild-type porin gene sequences from each were added to our custom SRST2 ARG-ANNOT database. Using our customized database, SSTAR identified truncations caused by internal stop codons in two major porin genes, *ompC* and *ompF*, in *E. coli* ST44 (Table 1). The T→A mutation at nucleotide position 246 in *ompC* creates a stop codon and thus a severely truncated protein that is likely nonfunctional. A 1-nucleotide deletion at position 193 in *ompF* resulted in a frameshift mutation and creation of a stop codon nearby, thereby possibly resulting in a lack of functional OmpF in this isolate. This inferred loss of porin function combined with the presence of *bla*_CTX-M-15_ likely explains the carbapenem resistance phenotype in this organism (Table 2).

**TABLE 2:** Observed MICs for multidrug-resistant *K. pneumoniae* ST437 and *E. coli* ST44^a^

Antimicrobial	*K. pneumoniae* ST437		*E. coli* ST44	
	MIC (µg/ml)	Interpretation	MIC (µg/ml)	Interpretation
Doripenem	>8	R	4	R
Ertapenem	>8	R	>8	R
Imipenem	32	R	2	I
Meropenem	>8	R	8	R
Aztreonam	>64	R	>64	R
Cefepime	>32	R	>32	R
Cefotaxime	>64	R	>64	R
Ceftazidime	>128	R	>128	R
Ceftriaxone	>32	R	>32	R
Cefazolin	>8	R	>8	R
Cefoxitin	>16	R	>16	R
Ampicillin	>32	R	>32	R
Amoxicillin-clavulanic acid	>32	R	>32	R
Piperacillin-tazobactam	>128	R	>128	R
Colistin	0.5	NA	0.25	NA
Polymyxin B	0.5	NA	0.5	NA
Amikacin	>64	R	16	S
Gentamicin	>16	R	1	S
Tobramycin	>16	R	>16	R
Tetracycline	8	I	>32	R
Tigecycline	4	I	<0.5	S
Trimethoprim-sulfamethoxazole	>8	R	>8	R
Chloramphenicol	>16	R	16	I
Ciprofloxacin	>8	R	>8	R
Levofloxacin	>8	R	>8	R

^a^ R, resistant; I, intermediate; S, susceptible; NA, no breakpoint available.

The *K. pneumoniae* ST437 isolate carried mutated porin genes in addition to *bla*_NDM-5_ and *bla*_OXA-232_. Two in-frame insertion sequences were identified in *ompK37*, at positions 695 and 813, neither of which resulted in internal stop codons. The first insertion sequence generates an extra 4 amino acids in OmpK37 (H-Y-T-H) and the second one a stretch of 6 (S-S-T-N-G-G). The protein sequence similarity between the OmpK37 protein deposited in our modified ARG-ANNOT database and the one detected in *K. pneumoniae* ST437 was 95%. This modified porin sequence has previously been deposited in the nonredundant (NR) database at the NCBI under accession number WP_002902433 and is not annotated as nonfunctional. Four silent mutations were identified in *ompK35* (156T→C, 294G→A, 303G→A, and 786C→T) and are most likely not involved in protein folding or function.

### Comparison of results obtained with SSTAR using the ARG-ANNOT database and the ResFinder 2.1 Web service.

In order to test the performance of SSTAR, we compared its results with those of the popular online ResFinder 2.1 Web service. Both SSTAR/ARG-ANNOT and ResFinder 2.1 (https://cge.cbs.dtu.dk//services/ResFinder/) identified *bla*_NDM-5_, *bla*_SHV-12_, *bla*_OXA-232_, *bla*_TEM-1_, and *bla*_OXA-9_ genes in *K. pneumoniae* ST437. However, the SSTAR/ARG-ANNOT approach identified an additional beta-lactamase gene, *bla_ampH_*. This gene is present in the ARG-ANNOT database but not included in the ResFinder repository (Table 1).

The aminoglycoside resistance gene *aadA1* was not detected by SSTAR due to several gaps and mismatches between the *aadA1* sequence present in ARG-ANNOT and the potential *aadA1* gene present in the *K. pneumoniae* genome. In contrast, the ResFinder database contains numerous *aadA1* genes, one of which displayed 100% sequence similarity to a gene in our query genome. A similar phenomenon was observed for *fosA*, a gene conferring resistance to fosfomycin. There were several *fosA* genes included in the ResFinder database, one of which was 97.14% similar to our query sequence, but ARG-ANNOT contained only a single *fosA* gene, which displayed only 74% nucleotide similarity with the identified *fosA* gene from ResFinder.

ResFinder 2.1 identified *bla*_OXA9_ in *K. pneumoniae* ST437 as having 99.88% sequence similarity to the gene in its database but was not identified as truncated and therefore likely nonfunctional. However, when SSTAR/ARG-ANNOT was used, this previously described mutation was readily identified and highlighted for the user (24).

To further evaluate the SSTAR algorithm, we downloaded the SRST2 ResFinder database (https://github.com/katholt/srst2) and evaluated SSTAR/ResFinder against our *K. pneumoniae* ST437 isolate. The same AR gene repertoire was identified using the ResFinder database queried with SSTAR and the ResFinder 2.1 tool (Table 1). The inclusion of multiple gene alleles in the ResFinder database allowed SSTAR to annotate the *bla*_TEM-1_ gene as *bla*_TEM-1A_, whereas it was identified as a possible new variant when the ARG-ANNOT database with only a single *bla*_TEM-1_ allele was used as the reference.

When this approach was used with the *E. coli* ST44 genome, the only observed difference was the detection of *bla_ampC1_* and *bla_ampC2_*, two putative cephalosporinase genes present in the ARG-ANNOT database that are not in the ResFinder collection. *bla_ampH_*, a weak beta-lactamase, was also identified by SSTAR in *E. coli* ST44 when ARG-ANNOT was used as a reference, but not with the ResFinder database.

## DISCUSSION

SSTAR is an easy-to-use, customizable tool for the very rapid identification of genes and potential variants associated with antimicrobial resistance. In addition, it is stand-alone and contains an intuitive graphical user interface that needs only a few clicks from the user for creating a local BLASTN database, identifying AR genes, as well as porin genes, and presenting these to the user. The average processing time depends heavily on the fragmentation of the genome assembly. A single isolate assembled from high-throughput Illumina short reads, currently the most common and popular sequencing platform, takes approximately 6 s on a 2.3-GHz Intel Core I5 processor laptop with 8 GB of RAM. Genome assemblies containing one long contiguous stretch of DNA, like PacBio or RefSeq sequences, will require considerably longer processing times. The automatic translation of PNVs is convenient and needs only BLASTP verification against a protein database or the beta-lactamase repository at http://www.ncbi.nlm.nih.gov/projects/pathogens/submit_beta_lactamase.

SSTAR’s translation to protein sequence enables detection of potential new variants, truncated enzymes, and porins that otherwise would be missed by examining only to the nucleotide level. This functionality could also be used with chromosomal/nonacquired genes to find new variants that might be involved in conferring resistance, e.g., novel *gyrA* changes associated with fluoroquinolone resistance (25 – 27). The detection of truncated gene products is especially useful for interrogating carbapenem-resistant microorganisms that do not produce a carbapenemase, where loss or truncation of porins is an important contributor to resistance phenotypes. Even when organisms do produce carbapenemases, porins can have a major role in the phenotypic profile and pathogenic success of particular clones (28).

When an internal stop codon is identified, SSTAR generates an invalid FASTA entry for that gene and therefore forces the user to recognize the “error” and remove the entry from the multi-FASTA file before aligning genes with other AR genes for the detection of PNVs.

Currently available AR detection tools will show the sequence similarity to only the reference gene for *bla*_OXA-9_; however, the users are not notified about possible nonfunctional variants. SSTAR identifies an internal stop codon in the *bla*_OXA-9_ beta-lactamase gene, resulting in a nonfunctional protein (24). This additional level of information will be increasingly important as genomic information is used to predict organism resistance, as simple gene detection is insufficient to predict phenotypes.

Different AR databases can generate different results due to their included genes. For instance, SSTAR/ARG-ANNOT identified a possible new variant of *bla*_TEM-1_; however, when performing the same annotation analyses using SSTAR/ResFinder, we observed a *bla*_TEM-1A_ wild type. This is due to the fact that ResFinder contains several TEM-1 alleles, whereas ARG-ANNOT contains only one. Thus, it is important that users of these AR databases understand the scope, strengths, and limitations of each repository. By querying multiple databases or creating customized databases, users will gain a more complete understanding of the complement of acquired and well-characterized resistance genes in their isolates of interest. SSTAR/ARG-ANNOT also did not identify the *aadA1* and *fosA* genes that are located in the *K. pneumoniae* ST437 genome, whereas SSTAR/ResFinder was able to detect those genes because several alleles of both genes were present in the ResFinder database. Using up-to-date AR gene databases is therefore essential for accurately predicting resistance genes, and SSTAR facilitates this option.

This is not meant to imply that genomic data can at this point predict the complete phenotypic profile of an isolate. For instance, both SSTAR/ResFinder and SSTAR/ARG-ANNOT were not able to explain the phenotypic resistance to chloramphenicol or tetracyclines for the *K. pneumoniae* ST437 isolate or the fluoroquinolone resistance in *E. coli* ST44. This further emphasizes the need for regular updating of AR databases as new genes are described and customization to include intrinsic mechanisms that strongly correlate with phenotype. Our tool is flexible and can easily be used with different enzyme databases, including any publicly accessible FASTA-formatted gene collection or custom database. When databases other than SRST2 ARG-ANNOT are used, each FASTA header needs to be structured with the CD-HIT-clustered SRST2 sequence header naming structure, which is described in our online manual (https://github.com/tomdeman-bio/Sequence-Search-Tool-for-Antimicrobial-Resistance-SSTAR-).

The SSTAR software is intended for biologists and clinical workers who want to investigate acquired antimicrobial resistance in bacterial genomes. Execution of SSTAR requires only two additional programs for its operation, BLAST+ and Java. This should make usage and installation easy for a broad audience with a limited bioinformatics background. In addition, SSTAR’s capacity for protein translation and identifying mutations that result in premature stop codons is unique among commonly available tools for resistance gene detection and thus provides the user with additional information about genotype-phenotype correlation that might otherwise be missed.

## MATERIALS AND METHODS

### Database selection.

**FIG 3:**
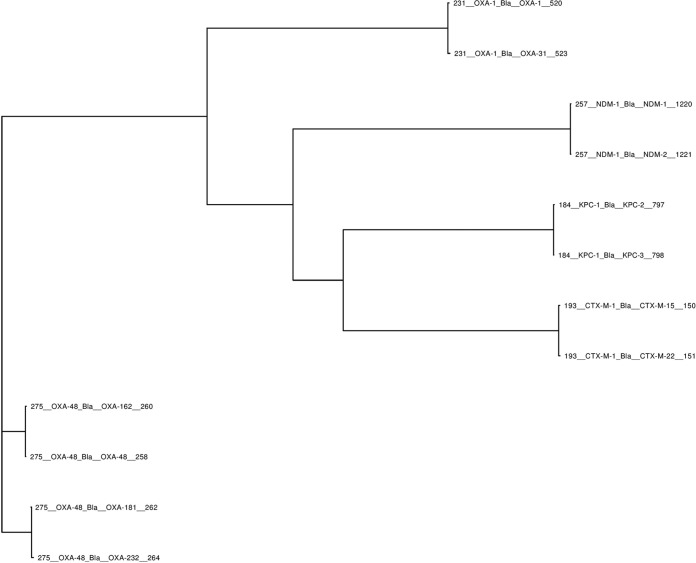
Several gene groups and associated alleles designated by SRST2 ARG-ANNOT. Each group is assigned a unique number at the beginning of the sequence header; e.g., *bla*_OXA-48_ alleles start with 275, and *bla*_KPC_ alleles start with 184, etc.

SSTAR can accept any database collection of resistance genes as a comparator for subsequent analysis. For this study, we selected the AR database from ARG-ANNOT, an extensive and curated collection of genes conferring resistance to different antibiotic classes, including aminoglycosides, beta-lactams, fosfomycin, fluoroquinolones, glycopeptides, macrolides, lincosamides, streptogramins, phenicols, rifampin, sulfonamides, tetracyclines, and trimethoprim. A slightly modified version of this database was downloaded from the SRST2 GitHub repository at https://github.com/katholt/srst2/. The AR gene sequences in this version are clustered by means of CD-HIT (29) into gene groups that share >80% sequence similarity. The sequence headers are formatted so that each clustered gene group has a unique number assigned to it. For instance, all New Delhi metallo-beta-lactamase (NDM) alleles fall within the same gene group and therefore start with the same number (i.e., 257). The OXA-type beta-lactamase family, which consists of more-diverse gene groups, contains multiple gene clusters, and each cluster has its own unique number (Fig. 3). SSTAR analyzes each group separately.

We manually excluded *tetR* from the database since it is a regulatory gene and not a resistance gene and *bla*_OXA-30_ because it is 100% identical to *bla*_OXA-1_ (30) (http://www.lahey.org/Studies). We also included several wild-type major outer membrane porin sequences from *K. pneumoniae* (*ompK35* [CP011313], *ompK36* [FJ577673], and *ompK37* [KC195784]), *E. coli* (*ompC* [HQ330974] and *ompF* [CP005930]), *Enterobacter cloacae* (*ompC* [AJ316539] and *ompF* [HM565105]), and *Enterobacter aerogenes* (*omp35* [AY487904] and *omp36* [AF336098]).

For comparison, we also used SSTAR to interrogate the genomes in this study against a CD-HIT-clustered version of the ResFinder repository (https://github.com/katholt/srst2/). The complete SSTAR package, along with both databases described above, are available at https://github.com/tomdeman-bio/Sequence-Search-Tool-for-Antimicrobial-Resistance-SSTAR-.

### Strains evaluated.

The *K. pneumoniae* ST437 and *E. coli* ST44 strains evaluated in this study were isolated from sputum and blood, respectively. These isolates were recognized as being unusually resistant and thus were submitted to the CDC for reference antimicrobial susceptibility testing (AST). AST was performed using the reference broth microdilution method according to the Clinical and Laboratory Standards Institute methodology (31) (Table 2). Additional molecular screening using real-time PCR for the detection of genes encoding KPC, NDM, and OXA-48-like carbapenemases was performed on each isolate as well. Whole-genome sequencing of both specimens, using a MiSeq benchtop sequencer (Illumina, San Diego, CA), was the final step in the process. Sequencing reads were then assembled by means of SPAdes 3.1 (32) and CLC Genomics Workbench v. 7.0.4 (CLC Bio, Aarhus, Denmark). Average genome coverage was 47-fold for *K. pneumoniae* and 122-fold for *E. coli*.

### Nucleotide sequence accession numbers.

The *K. pneumoniae* ST437 and *E. coli* ST44 genome sequences have been deposited in DDBJ/EMBL/GenBank under the accession numbers LART00000000 and LAXC00000000, respectively. The versions used in this paper are LART01000000 and LAXC01000000.
